# Significant loss of retinal nerve fibre layer and contrast sensitivity in people with well controlled HIV disease: implications for aging with HIV

**DOI:** 10.3389/fopht.2023.1251126

**Published:** 2023-12-15

**Authors:** Malinee Neelamegam, Nilani Nawi, Nor Syuhada Ahmad Bashah, Yap Siew Hwei, Nurul Syuhada Zulhaimi, Adeeba Kamarulzaman, Shahrul Bahyah Kamaruzzaman, Norlina Ramli, Reena Rajasuriar

**Affiliations:** ^1^ Department of Medicine and Centre for Excellence for Research in Acquired Immunodeficiency Syndrome (AIDS), University Malaya, Kuala Lumpur, Malaysia; ^2^ Department of Biostatistics and Epidemiology, School of Public Health, University of North Texas Health Science Center, Fort Worth, TX, United States; ^3^ University Malaya Eye Research Centre, Department of Ophthalmology, Faculty of Medicine, University Malaya, Kuala Lumpur, Malaysia; ^4^ Geriatrics Unit, Department of Medicine, Faculty of Medicine, University Malaya, Kuala Lumpur, Malaysia; ^5^ The Doherty Institute for Infection and Immunity, The University of Melbourne, Melbourne, VIC, Australia

**Keywords:** HIV, retina, contrast sensitivity, visual acuity, antiretroviral therapy

## Abstract

**Objective:**

Antiretroviral therapy has decreased the prevalence of retinal opportunistic infections in people living with HIV (PLWH). However, abnormalities in visual function are evident and may be associated with an early onset of aging in PLWH. In this study, we examined the Retinal Nerve Fibre Layer (RNFL) thickness and visual function in PLWH and HIV non-infected controls in Malaysia.

**Design:**

Cross-sectional study

**Methods:**

Two hundred and two (202) PLWH without retinal opportunistic infection and 182 age-matched, HIV seronegative individuals were enrolled. PLWH were recruited from the Infectious Disease clinic at the University Malaya Medical Centre. Controls were recruited among the hospital staff and community volunteers. RNFL thickness was measured with spectral domain optical coherence tomography (SDOCT). Visual functions include visual acuity using LogMAR chart and contrast sensitivity using Pelli- Robson Chart.

**Results:**

All PLWH (mean age 46.1 years ± 9.9 years) in the study were on ART and 61.2% had a CD4+ T-cell count more than 500 cell/μl. The mean visual acuity was similar between the two groups (LogMAR 0.05 vs. 0.07, p = 0.115). Contrast sensitivity was lower in PLWH compared to HIV seronegative individuals (1.90 vs 1.93, p = 0.032). RNFL thickness was significantly thinner in the temporal quadrant for PLWH compared to controls (68.89 μm vs 74.08 μm, p = 0.001).

**Conclusion:**

Changes in RNFL thickness and contrast sensitivity were seen in PLWH despite their relatively young age and well controlled HIV disease. The changes reflect structural and functional deficits, and could have long-term implications on their health trajectory.

## Introduction

Advancements in the field of HIV treatment has markedly altered the spectrum of HIV-related eye diseases and significantly lowered the prevalence of opportunistic ocular infections in people living with HIV (PLWH) (1). However, in the current treat-all era, life expectancy in PLWH is fast approaching that of the general population. As such, issues related to age-related changes have become prevalent and contribute to significant morbidity among PLWH. This includes comorbidities related to visual function, such as reduced contrast sensitivity, decreased colour vision and visual field defects (2–4). It has been postulated that HIV infection accelerates/accentuates the process of biological aging (5, 6). In the eye, these changes may be mediated by multiple aetiologies, including HIV-associated neuroretinal disorder (HIV-NRD), where degenerative changes occur in the retinal nerve fibre layer (RNFL)(7).

RNFL loss may affect long term visual function in PLWH including abnormal contrast sensitivity. This is important as abnormal contrast sensitivity can be more symptomatic than reduced visual acuity (8). Few studies to date have assessed changes in vision among PLWH especially in the context of well controlled HIV disease and from the low-middle income setting. In these settings, established risk factors for neuroretinal damage, such late presentation of disease with prolonged periods of uncontrolled viremia and immune activation, are relatively common (9, 10). Additionally, exposure to more toxic antiretroviral therapies with mitochondrial toxicity, also termed as D-drugs, are far more prevalent in PLWH in resource-limited settings. This may have a lasting impact on retinal nerve damage. In a case series reporting didanosine retinal toxicity in adults living with HIV, the authors postulated the pathomechanism suggestive of mitochondrial DNA damage (11).

This study aimed to examine the differences in RNFL thickness and contrast sensitivity among PLWH and age-matched HIV non-infected controls. We also set out to determine if any correlation exists between HIV- related parameters including treatment exposure and systemic biomarkers of immune activation with RNFL thickness. Correlates of RNFL thickness with cognitive function was also explored.

## Methods

### Design & setting

This is a cross sectional study conducted in the University Malaya Medical Centre from July 2014 till June 2016. PLWH were recruited from the infectious disease clinic during their routine follow up. Ophthalmic assessments and retinal imaging for RNFL thickness was done in the ophthalmology clinic. All study participants provided informed consent prior to recruitment and the study protocol was approved by the University of Malaya Research Ethic Committee (MEC 20151-937).

Inclusion criteria for PLWH were as follows; participants aged 25 years and above and receiving ART with undetectable HIV RNA (<50copies/ml) for at least 1 year prior to recruitment. Those with media opacities (i.e cataract, corneal scarring), known ocular pathology (i.e retinal haemorrhage, retinopathy, uveitis), history of previous retinal opportunistic infection and myopia equal or more than 2 diopters were excluded. A control group of age-matched HIV seronegative individuals were recruited among volunteers in the community and hospital. All control participants underwent a rapid HIV screening test to confirm seronegativity prior to recruitment.

### Data collection

Data on age, sex and ethnicity as well as comorbidities were collected from all subjects and controls through clinical examination and medical chart review. Additional information collected for PLWH included current CD4 counts, viral loads, ART duration and current and previous ART regimen. Prior exposure to D-drugs were defined as exposure to nucleoside reverse transcriptase inhibitors (NRTIs) previously shown to be associated with mitochondrial toxicity (ddI (didanosine), ddC (zalcitabine), d4T (stavudine) and ZDV (zidovudine)). All participants were subjected to a detailed ophthalmological assessment. Visual acuity was assessed using the LogMAR chart. Contrast sensitivity was measured in ambient illumination using the Pelli-Robson chart as recommended by the manufacturer. RNFL thickness measurements was performed using the Spectral Domain Cirrus OCT model 4000 (Carl Zeiss Meditec, Dublin, CA, USA). The scans were obtained by a trained technician and only scans of good quality (score of more than 7 out of 10 with no movement artefacts or distortions) were used for analysis. The measurement of the retinal nerve fibre layer was obtained as three-dimensional cube data using the Optic Disc Cube 200 x 200 scan. The software automatically locates the disc centre which is the circumpapillary centre (3.4mm in diameter) and calculates the RNFL thickness from the data. The RNFL measurement output is divided into four quadrants occupying 90° each, namely the superior, inferior, nasal and temporal quadrants. The parameters evaluated include the global RNFL average and RNFL thickness in each quadrant. Although both eyes were examined, only measurements from the right were used for analysis. Data from the left eye was used if the data for the right eye was excluded for reasons mentioned under the exclusion criteria or yielded low quality scans.

### Systemic biomarkers of immune activation

All participants also provided 10mls of whole blood collected in EDTA vacutainers for biomarker analysis. Immunophenotyping on whole blood for CD4+ and CD8+ T-cell activation markers (HLA-DR+ and CD38+) was conducted using protocols previously optimised.(12). Data was acquired on a FACS Canto II and analysed using FACS Diva (BD Biosciences, USA). The marker of monocyte activation, soluble CD14+ (sCD14+) was measured in plasma samples using an ELISA platform (R&D Systems, Minneapolis, MN) while indolamine 2,3 dehydrogenase (IDO) activity, a marker of interferon-driven immune activation was estimated by quantifying the ratio of plasma kynurenine to tryptophan using LCMS/MS as previously described (13). These markers were selected as they have been shown to remain persistently elevated in PLWH on ART and/or associated with mortality and non-AIDS defining events (14, 15).

### Statistical analyses

Demographic and clinical characteristics of study participants were examined using t-tests and Fisher’s exact test. T-test was used to examine the difference in visual acuity, contract sensitivity and RNFL among PLWH and controls. Linear regression models were used to assess the association between HIV parameters and contrast sensitivity. Generalized Linear Models (GLM) were used to assess the association between key factors and RNFL thickness in PLWH and controls. Three statistical models, representing an unadjusted model (Model 1), a partially adjusted model for demographic variables (Model 2) and a fully adjusted model, additionally adjusting for comorbidities and HIV-parameters (Model 3) were used to assess these associations. The association between biomarkers and RNFL thickness was assessed using models adjusted for age, sex, race, diabetes, and hypertension. To minimize Type 1 error due to multiple comparisons, statistical significance was maintained at 0.01. Data analysis was completed in SAS (v.9.4, SAS Institute Inc., Cary, North Carolina).

## Results

### Population characteristics

Two hundred and two (202) PLWH and 182 age-matched HIV seronegative controls were recruited in this study. The demographic characteristics of study participants are presented in **Table 1**. Participants were predominantly male, 162 (80.2%) PLWH and 119 (65.4%) controls. Mean age of PLWH was 46.1 years ± 0.7 and 44.5 years ± 0.8 in controls. Most PLWH in the study had CD4+ T-cell counts of more than 500 cells/μl (61.2%). The majority had received ART for more than two years with 73.8% on non-nucleoside reverse transcriptase inhibitor (NNRTI) regimen. PLWH had higher levels of immune activation markers.

**Table 1:** Demographic and clinical characteristics of individuals living with HIV and seronegative individuals. .

Characteristics		HIV positive n = 202	Control n = 182
Gender, n (%)***	Male	162 (80.2)	119 (65.4)
Age, mean (SD)		46.1 (9.9)	44.5 (11.3)
Race, n (%)**	Malay	38 (18.8)	60 (33.0)
	Chinese	142 (70.3)	103 (56.6)
	Indian	19 (9.4)	18 (9.9)
	Others	3 (1.5)	1 (0.5)
Diabetes, n (%)		25 (12.7)	6 (6.3)
Hypertension, n (%)		78 (39.4)	38 (40.9)
Smoking status, n (%)***	Current smoker Former smoker	53 (31.5) 23 (13.7)	12 (12.6) 4 (4.2)
Montreal Cognitive Assessment (MoCA) score, mean (SD)**		25.41 (3.07)	26.12 (2.29)
K/T ratio, mean (SD)**		0.031 (0.013)	0.026 (0.008)
CD4+ T-cell count >500 (cells/μl) Baseline CD4+ T-cell count (cells/µl)		123 (61.2)	
	
		152.9 (136.6)	
	
Prior exposure to ddI/ddC/d4T/ZDV, n (%)		140 (69.3%)	
AIDS Defining Illness, n (%)		73 (42.7)	
CD4:CD8 ratio		0.72 (0.43)	
ART duration, n (%)	<24 months	28 (14.4)	
	24-48 months	41 (21.0)	
	49-72 months	38 (19.5)	
	73-120 months	44 (22.6)	
	>120 months	44 (22.6)	
ART Type, n (%)	NNRTI-based	149 (73.8)	
	PI-based	23 (11.4)	
	INSTI-based	2 (1.0)	
	Other	1 (0.5)	
LogMAR acuity, mean (SD)		0.05 (0.07)	0.07 (0.09)
Contrast sensitivity, mean, (SD)*		1.90 (0.12)	1.93 (0.13)
RNFL Thickness, µm (SD) Average Superior Nasal Inferior Temporal***		95.6 (11.6) 119.4 (20.3) 124.9 (10.2) 69.3 (19.9) 68.9 (13.1)	95.9 (10.2) 118.6 (17.3) 124.6 (12.2) 69.2 (19.2) 74.1 (13.5)

*indicates significance at P<0.05 **Indicates significance at P<0.01 ***indicates significance at P<0.001.

RNFL, Retinal Nerve Fibre Layer; ddI/ddC/d4T/ZDV, didanosine/zalcitabine/stavudine/zidovudine; NNRTI, Non-nucleoside reverse transcriptase inhibitor; PI, Protease inhibitor; INSTI, Integrase strand transfer inhibitor.

### Visual acuity and contrast sensitivity

There was no significant difference in visual acuity among PLWH and controls (**Table 1**). The mean LogMAR acuity was 0.05 ± 0.07 in PLWH and 0.07 ± 0.09 in controls (p=0.115). Contrast sensitivity scores were lower among PLWH compared to controls (1.90 ± 0.12 vs 1.93 ± 0.13, p<0.05). However, when explored further, among PLWH in our study, contrast sensitivity was not associated with HIV-related parameters including current and baseline CD4+ T-cell count, CD4:CD8 ratio, prior exposure to ddI/ddC/d4T/ZDV and duration on ART (**Table 2**).

**Table 2:** Factors associated with RNFL thickness in people living with HIV and HIV non-infected individuals.

Factors	Average RNFL β estimate (95% CI)		Superior Quadrant β estimate (95% CI)		Nasal Quadrant β estimate (95% CI)		Inferior Quadrant β estimate (95% CI)		Temporal Quadrant β estimate (95% CI)	
	PLWH	Control	PLWH	PLWH	PLWH	Control				
Age	-0.16(-0.32,-0.01)	-0.15 (-0.29, -0.02)	-0.30 (-0.57, -0.02)*	-0.29 (-0.52, -0.06)*	-0.03 (-0.17, 0.11)	0.04 (-0.13, 0.20)	-0.22 (-0.50, 0.05)	-0.08 (-0.33, 0.18)	-0.13 (-0.31, 0.06)	-0.25 (-0.42, -0.07)*
Female	2.93 (-0.98, 6.84)	1.81 (-1.28, 4.91)	3.46 (-3.37, 10.30)	2.06 (-3.19, 7.30)	0.67 (-2.82, 4.16)	-1.54 (-5.27, 2.19)	4.84 (-1.86, 11.55)	-0.52 (-6.42, 5.37)	3.02 (-1.48, 7.52)	3.59 (-0.35, 7.52)
Race Chinese Indian	0.10 (-4.13, 3.92) 6.27 (0.07, 12.47)	0.91 (-2.38, 4.19) -1.49 (-6.76, 3.78)	-3.35 (-10.39, 3.70) 8.46 (-2.38, 19.30)	0.47 (-5.11, 6.04) -3.98 (-12.92, 4.96)	2.12 (-1.48, 5.72) 5.64 (0.10, 11.19)	-2.28 (-6.24, 1.68) 2.48 (-3.87, 8.84)	-1.86 (-8.77, 5.05) 9.52 (-1.11, 20.16)	-0.77 (-7.03, 5.49) -5.02 (-15.06, 5.02)	0.85 (-3.79, 5.49) -0.16 (-7.30, 6.99)	8.45 (4.29, 12.63)** -0.25 (-6.95, 6.44)

PLWH, People living with HIV.

*indicates significance at P<0.01 **indicates significance at P<0.001. Model adjusted for age, gender and race.

### Retinal nerve fibre layer

Average RNFL thickness and the RNFL thickness of specific quadrants in PLWH and controls are presented in **Table 1**. There was a significant difference in the temporal quadrant RNFL thickness among PLWH and controls (68.89 μm vs 74.08 μm, p < 0.001).

**Figure 1:**
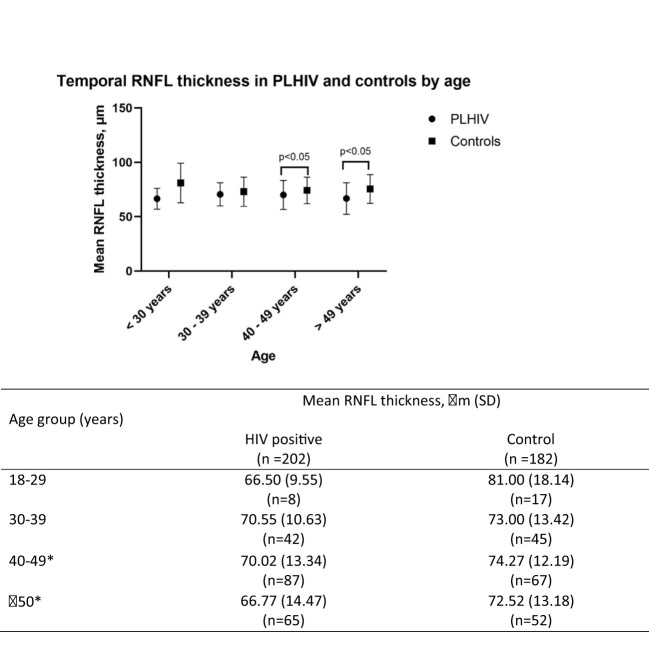
Temporal RNFL thickness between HIV and control group by age group. *indicates significance at P<0.05. SD, Standard deviation; RNFL, retinal nerve fibre layer.

**Figure 1** presents the difference in the average RNFL thickness in the temporal quadrant in PLWH and controls, according to age groups. While there was no significant difference in the average temporal RNFL thickness in the 2 groups, among those aged 39 years and younger, a significant difference is seen in those aged 40 years and above. Compared to controls, average temporal RNFL thickness was significantly lower among PLWH aged 40 to 49 years and those 50 years and older.

Findings from multivariate analysis of factors associated with RNFL thickness among PLWH and controls for Model 2 and Model 3 are presented in **Tables 2**, **3** respectively. Both in PLWH and controls, diabetes emerged as a predominant factor significantly associated with RNFL thickness. In PLWH, diabetes was significantly associated with thinner average RNFL (β =-7.66, 95% CI -13.42, -1.89, p<0.01) and inferior quadrant RNFL (β= -11.99, 95% CI -21.75, -2.22, p<0.001) and the temporal quadrant (β= -9.35, 95% CI -16.04, -2.66, p<0.001). Among controls, diabetes was significantly associated with thinner average RNFL (β =-15.57, 95% CI -25.31, -5.83, p<0.001), and temporal quadrant RNFL (β= -12.71, 95% CI -23.24, -2.18, p<0.01).

**Table 3:** Factors associated with RNFL thickness in people living with HIV and HIV seronegative individuals.

Factors	Average RNFL β estimate (95% CI)		Superior Quadrant β estimate (95% CI)		Nasal Quadrant β estimate (95% CI)		Inferior Quadrant β estimate (95% CI)		Temporal Quadrant β estimate (95% CI)		
	PLWH	Control	PLWH	PLWH	PLWH	Control					
Age	-0.11 (-0.21, 0.19)	-0.15 (-0.35, 0.01)	-0.41(-0.83, 0.01)	-0.29 (-0.64, 0.05)	0.10 (-0.11, 0.32)	-0.07 (-0.32, 0.17)	0.11 (-0.23, 0.45)	-0.10 (-0.49, 0.29)	-0.05 (-0.29, 0.18)	-0.10 (-0.33, 0.13)
Female	2.79 (-1.27,6.86)	3.12 (-2.19, 8.43)	3.00 (-4.33, 10.32)	4.66 (-4.32, 13.64)	0.69 (-3.05, 4.43)	1.01 (-5.40, 7.41)	2.18 (-4.10, 8.47)	2.96 (-7.21, 13.12)	3.73 (-0.58, 8.03)	0.23 (-5.50, 5.96)
Race Chinese Indian	0.44 (-3.81, 4.69) 4.68 (-1.97, 11.34)	0.20 (-4.70, 5.10) 4.49 (-5.08, 14.06)	-1.71 (-9.37, 5.96) 8.09 (-3.89, 20.07)	-0.03 (-8.32, 8.26) 2.07 (-14.12, 18.27)	1.49 (-2.42, 5.41) 4.50 (-1.62, 10.61)	-3.17 (-9.09, 2.74) 2.52 (-9.03, 14.08)	0.02 (-6.58, 6.62) 10.42 (0.48, 20.36)	-2.77 (-12.15, 6.61) 6.37 (-11.96, 24.69)	1.03 (-3.49, 5.55) -1.12 (-7.93, 5.70)	8.25 (2.99, 13.51)* 6.14 (-4.14, 16.41)
Diabetes	-7.66 (-13.42, - 1.89)**	-15.57 (-25.31, -5.83)**	-2.97 (-12.64, 6.70)	-20.48 (-36.96, -3.99)	0.69 (-4.24, 5.63)	-9.98 (-21.75, 1.78)	-11.99 (-21.75, -2.22)**	-18.70 (-37.35, -0.04)	-9.35 (-16.04, -2.66)**	-12.71 (-23.24, -2.18)*
Hypertension	3.36 (-0.11, 6.84)	1.62 (-2.92, 6.15)	5.40 (-0.85, 11.66)	0.46 (-7.21, 8.14)	0.25 (-2.95, 3.44)	1.81 (-3.67, 7.28)	-0.45 (-5.81, 4.92)	0.16 (-8.52, 8.85)	4.14 (0.46, 7.82)	0.43 (-4.44, 5.31)
Cognition (MoCA)	-0.09 (-0.62, 0.45)		-0.65 (-1.62, 0.32)	-0.19 (-1.86, 1.48)	-0.01 (-0.51, 0.48)	-0.66 (-1.85, 0.53)	-0.32 (-1.17, 0.53)	0.79 (-1.10, 2.68)	0.86 (0.27, 1.44)*	0.96 (-0.10, 2.02)
CD4/CD8 ratio	4.56 (-0.10, 9.23)		5.49 (-2.91, 13.89)		0.29 (-3.17, 3.75)		-0.12 (-6.16, 5.92)		-1.43 (-5.57, 2.71)	
Duration on cART	-0.002 (-0.04, 0.03)		-0.03 (-2.91, 13.89)		-0.01 (-0.04, 0.01)		0.02 (-0.04, 0.08)		0.04 (0.01, 0.07)	
Baseline CD4	-0.01 (-0.03, 0.001)		-0.02 (-0.05, 0.003)		-0.01 (-0.03, 0.002)		-0.02 (-0.04, 0.01)		-0.001 (-0.02, 0.02)	
Prior exposure to ddI/ddC/d4T/ZDV and CD4:CD8 ratio	1.06 (-2.05, 5.68)		1.38 (-3.49, 6.99)		2.31 (-3.33, 4.91)		1.23 (-2.75, 3.94)		1.54 (-3.32, 5.31)	

PLWH, People living with HIV; MoCA, Montreal Cognitive Assessment; *indicates significance at P<0.01 **indicates significance at P<0.001. Model adjusted for demographics, comorbidities and HIV parameters (Current CD4, Baseline CD4, Duration on cART, Prior exposure to ddI/ddC/d4T/ZDV and CD4:CD8 ratio).

In PLWH, individuals with higher MoCA scores, indicating lesser cognitive impairment, had thicker temporal quadrant RNFL. (β=0.86, 95% CI 0.27, 1.44, p<0.01). However, HIV-related parameters, including baseline CD4, CD4:CD8 ratio, prior exposure to ddI/ddC/d4T/ZDV and duration on ART, were not significantly related with RNFL thickness.

### Biomarkers of immune activation and senescence and RNFL thickness

**Table 4** presents the adjusted association of relevant biomarkers and RNFL thickness among PLWH and controls. Among PLWH, CD8+ T-cell activation measured as the proportion co-expressing CD38+ and HLA-DR+ was associated with average RNFL thickness, as well as RNFL thickness in the superior and inferior quadrant. Compared to PLWH in the lowest quartile of CD8+ T-cell activation, PLWH in the third quartile paradoxically had significantly higher RNFL thickness (Average RNFL: β=6.32, 95% CI 1.71, 10.94, p<0.01, Superior Quadrant: β=13.38, 95% CI 5.08, 21.68, p<0.01, Inferior Quadrant: β=11.18, 95% CI 3.15, 19.22, p<0.01).

**Table 4:** Biomarkers associated with RNFL thickness in people living with HIV and HIV seronegative individuals. .

Potential Biomarkers	Average RNFL β estimate (95% CI)		Superior Quadrant β estimate (95% CI)		Nasal Quadrant β estimate (95% CI)		Inferior Quadrant β estimate (95% CI)		Temporal Quadrant β estimate (95% CI)	
	PLWH	Control	PLWH	Control	PLWH	Control	PLWH	Control	PLWH	Control
CD4+CD38+ Quartile 2 Quartile 3 Quartile 4	1.51 (-3.05,6.07) 5.18 (0.89, 9.47) 0.46 (-3.81, 4.74)	2.79 (-2.92, 8.51) 2.32 (-3.61, 8.25) 5.48 (-0.35, 11.31)	-1.56 (-9.70, 6.59) 4.09 (-3.57, 11.76) -2.84 (-10.48, 4.80)	5.14 (-4.78, 15.05) -0.27 (-10.56, 10.02) 10.68 (0.57, 20.79)	-0.59 (-5.06, 3.88) 0.01 (-4.20, 4.21) -4.17 (-8.37, 0.02)	-0.44 (-6.98, 6.10) 3.79 (-3.00, 10.57) 5.34 (-1.33, 10.01)	2.24 (-5.86, 10.33) 5.65 (-1.96, 13.27) 2.27 (-5.32, 9.86)	6.48 (-3.82, 16.79) 2.72 (-7.97, 13.41) 1.20 (-9.32, 11.71)	3.94 (-1.64, 9.52) 8.83 (3.58, 14.08)** 5.32 (0.08, 10.55)	-0.18 (-6.54, 6.19) 4.98 (-1.72, 11.69) 1.48 (-5.01, 7.96)
CD4+CD38+HLA-DR+ Quartile 2 Quartile 3 Quartile 4	2.50 (-3.10, 8.09) 1.87 (-3.59, 7.32) 2.09 (-3.18, 7.34)	2.96 (-2.05, 7.98) -1.39 (-7.05, 4.28) 6.01 (-5.89, 17.91)	2.03 (-7.98, 12.05) 2.90 (-6.86, 12.66) 6.63 (-2.79, 16.05)	3.13 (-5.50, 11.76) -1.48 (-11.22, 8.26) 1.95 (-18.52, 22.42)	-0.96 (-6.50, 4.58) -0.17 (-5.57, 5.23) -1.73 (-6.94, 3.48)	4.15 (-1.51, 9.82) -0.91 (-7.31, 5.48) 5.56 (-7.88, 18.99)	6.35 (-3.45, 16.16) 3.10 (-6.45, 12.66) 4.85 (-4.37, 14.07)	6.83 (-2.03, 15.70) 0.80 (-9.21, 10.81) 9.65 (-11.39, 30.68)	3.09 (-3.71, 9.89) -0.55 (-7.17, 6.07) -1.04 (-7.43, 5.36)	2.48 (-2.92, 7.87) 1.57 (-4.66, 7.81) 5.75 (-7.02, 18.51)
CD8+CD38+ Quartile 2 Quartile 3 Quartile 4	1.06 (-3.77, 5.89) 0.91 (-3.42, 5.24) -1.39 (-5.87, 3.10)	7.98 (2.58, 13.37)* 4.68 (-1.49, 10.84) 9.49 (2.93, 16.04)*	0.01 (-8.62, 8.64) 2.00 (-5.74, 9.75) -3.78 (-11.80, 4.24)	10.23 (1.24, 19.22) 7.48 (-2.80, 17.76) 10.27 (8.34, 30.20)**	0.61 (-4.09, 5.31) -4.19 (-8.41, 0.02) -2.87 (-7.23, 1.49)	3.69 (-2.61, 9.99) 0.80 (-6.41, 8.00) 3.22 (-4.44, 10.88)	0.97 (-7.50, 9.44) 4.49 (-3.11, 12.10) 3.09 (-4.78, 10.97)	6.95 (-2.84, 16.75) 5.93 (-5.28, 17.13) -8.56 (-34.16, 17.04)	1.55 (-4.38, 7.48) 0.73 (-4.59, 6.05) -1.75 (-7.26, 3.76)	11.63 (6.06, 17.20)** 5.94 (-0.38, 12.26) 10.36 (3.65, 17.08)*
CD8+CD38+HLA-DR+ Quartile 2 Quartile 3 Quartile 4	0.26 (-4.60, 5.12) 6.32 (1.71, 10.94)* 0.41 (-4.13, 4.94	-1.08 (-6.20, 4.05) 2.94 (-2.68, 8.56) 6.84 (-1.79, 1.47)	2.85 (-5.89, 11.59) 13.38 (5.08, 21.68)* 5.37 (-2.79, 13.52)	-3.35 (-12.14, 5.45) 9.31 (-0.35, 18.97) 7.86 (-6.95, 22.68)	0.13 (-4.78, 5.04) -1.43 (-6.09, 3.24) -3.27 (-7.86, 1.30)	0.01 (-5.83,5.85) 2.67 (-3.75, 9.09) 0.09 (-9.75, 9.94)	-2.24 (-10.71, 6.22) 11.18 (3.15, 19.22)* 3.17 (-4.72, 11.06)	-0.34 (-9.32, 8.64) 1.24 (-8.63, 11.10) 13.11 (-2.01, 28.24)	-2.34 (-8.44, 3.76) 0.54 (-5.25, 6.33) -4.28 (-9.97, 1.41)	-1.25 (-6.90, 4.41) -2.47 (-8.73, 3.79) 4.72 (-4.82, 14.25)
sCD14, 10 -6 Quartile 2 Quartile 3 Quartile 4	1.29 (-3.85, 6.44) -0.54 (-5.23, 4.15) 0.31 (-4.33, 4.95)	-2.43 (-7.27, 2.40) -0.74 (-6.95, 5.47) -0.97 (-9.02, 7.09)	-5.01 (-14.18, 4.16) -4.67 (-13.03, 3.69) -7.30 (-15.57, 0.97)	-1.60 (-9.66, 10.94) 0.64 (-9.66, 10.94) 2.84 (-10.52, 16.21)	0.29 (-4.72, 5.29) 0.52 (-4.04, 5.09) -0.36 (-4.88, 4.15)	-0.60 (-6.43, 5.23) 0.64 (-6.85, 8.13) -0.77 (-10.49, 8.96)	11.27 (2.65, 19.89)* 7.40 (-0.46, 15.26) 7.20 (-0.58, 14.97)	-0.51 (-9.82, 8.79) 4.07 (-7.87, 16.01) -8.05 (-23.54, 7.45)	-0.78 (-6.84, 5.30) -4.87 (-10.40, 0.66) -1.18 (-6.66, 4.29)	-0.83 (-6.07, 4.42) -1.98 (-8.66, 4.69) -0.67 (-9.34, 7.99)
K/T ratio Quartile 2 Quartile 3 Quartile 4	1.95 (-2.71, 6.62) 1.32 (-3.33, 5.97) 3.74 (-0.85, 8.34)	-0.28 (-5.91, 5.34) -3.92 (-10.42, 2.57) -4.74 (-12.13, 2.65)	1.73 (-6.67, 10.14) 5.13 (-3.24, 13.51) 7.60 (-0.68, 15.87)	-3.07 (-12.78, 6.64) -6.18 (-17.38, 5.03) -2.14 (-14.90, 10.62)	4.89 (0.40, 9.38) 3.32 (-1.16, 7.79) 4.81 (0.38, 9.23)	-6.53 (-12.97, -0.09) -7.97 (-15.40, -0.54) -11.07 (-19.53, -2.61)*	3.92 (-3.85, 11.70) 2.80 (-4.95, 10.55) 5.04 (-2.63, 12.70)	6.98 (-3.15, 17.120 0.35 (-11.34, 12.05) -5.17 (-18.49, 8.14)	-3.91 (-9.34, 1.52) -3.11 (-8.53, 2.29) -1.55 (-6.90, 3.80)	-1.17 (-7.40, 5.07) -10.06 (-17.34, -2.78)* -8.02 (-16.21, 0.18)

*indicates significance at P<0.01 **indicates significance at P<0.001. Models adjusted for demographics (age, gender and race), diabetes and hypertension.

Among controls, CD8 + T-cell activation measured as the proportion expressing CD38+ was also associated with RNFL thickness, in addition to K/T ratio. Compared to individuals in the lowest quartile with these markers, individuals in the second and fourth quartile had significantly higher RNFL thickness, similar to the association found among PLWH. However, an increased K/T ratio, denoting increased interferon-driven immune activation, was associated with reduced RNFL in the nasal and temporal quadrant of controls. Compared to individuals in the lowest quartile of K/T ratio, individuals in higher quartiles of K/T ratio had reduced RNFL thickness (Nasal Quadrant (4^th^ quartile): β=-11.07, 95% CI -19.53, -2.61, p<0.01, Temporal Quadrant (3^rd^ quartile): β=-10.06, 95% CI -17.34, -2.78, p<0.01). We found an inconsistent association between RNFL thickness and levels of sCD14 and CD4+ T-cell activation markers across quartiles in some quadrants.

## Discussion

Few studies have examined ocular dysfunction in PLWH and especially from the LMIC setting where drivers of immune activation and coinfections may persist despite treatment and modulate the pathogenesis of age-associated organ decline. This study shows that HIV infection is associated with significant RNFL thinning in the absence of opportunistic retinal infection and well controlled HIV disease in a relatively young cohort. A reduction of up to one line in contrast sensitivity is also seen in PLWH. This is in agreement with a similar study by Pathai et al. who additionally found increasing duration on ART to be associated with RNFL thinning (1). In this study however, we did not find any association with ART duration or any other HIV-related parameters including the exposure of D-drugs, which has not been explored in previous studies. Conversely, diabetes, which is a non-AIDS related morbidity of increasing importance among PLWH on long-term ART, was found to be an independent risk factor associated with RNFL loss across multiple quadrants. Our findings demonstrate the interplay of risk factors for neuro-retinal disorders in an aging cohort of PLWH, where contemporary issues of multimorbidity overlap with legacy effects similar to that observed with cognitive issues among PLWH.

Our study supports existing data which shows that HIV infection results in abnormalities of the RNFL as evidenced by ocular imaging (3, 4, 16–19). Studies in the adult population in both USA and South Africa show that these structural abnormalities also manifest functionally as a reduction in contrast sensitivity but not visual acuity (1, 4). Other studies have demonstrated that these functional deficits are demonstrable in formal automated visual field tests (20, 21). We did not perform visual field testing in our study. However, the significant reduction in contrast sensitivity in PLWH serves as a good reflection of reduced visual function. Kozak et al. demonstrated significant outer retinal dysfunction in HIV positive retinae using a combination of retinal imaging and transcriptomic techniques (18). Their findings of partial loss of rod and cone photoreceptors found in cadaveric eyes of HIV positive patients who did not have opportunistic retinal infections likely explains the loss of contrast sensitivity function.

RNFL thickness was shown in this study to be reduced with increasing age in both groups. This is consistent with the findings that RNFL thinning occurs as part of the ageing process (22, 23). However the HIV positive group had significantly thinner RNFL compared to their age matched controls, except for those in age 30-39 years (70.55μm vs 73μm, p=0.35). Based on these findings, it appears that age related RNFL thinning occurs sooner in PLWH compared to uninfected controls. This finding, combined with reduced contrast sensitivity is collectively known as part of HIV associated neuroretinal disorders (HIV-NRD) (24). The spectrum of changes in the HIV-NRD is postulated to be mediated by several processes such as micro-vasculopathy, (25–27) chronic inflammation (10) or direct damage of neural tissue by HIV or ART (9, 28). HIV-NRD is implicated by some researchers to play a big role in accelerated biological aging associated with HIV infection (24, 29). Declines in visual function (as a proxy to RNFL thinning) are easier to perform and can be done by non-specialist healthcare workers even in the community settings. As visual impairment has repeatedly been shown to be associated with poor cognitive aging, visual function in adults aging with HIV can be used as proxy indicator to identify individuals requiring referral for cognitive assessments in resource limited settings, where the availability of cognitive screening tools and technicians trained to perform them are limited. Individuals of Chinese ethnicity are over-represented in our study population, possibly due to the location where the study was conducted (urban tertiary medical centre). This is a limitation of the study as it does not demographically reflect the Malaysian population.

Our findings on the association between diabetes and reduced RNFL thickness further corroborates findings of previous studies (30, 31). RNFL thinning is the earliest neurodegenerative change that is noted with diabetic retinopathy (32). While diabetic retinopathy has long been linked to vascular changes, studies have reported that retinal neurodegeneration in diabetics precede microvascular changes in the eye (33). This is of particular concern among PLWH residing in the LMIC settings as HIV infection is associated with an increased risk of developing diabetes (34, 35).

In multivariate analysis, changes in RNFL thickness showed no relationship with current and baseline CD4+ T-cell counts, CD4:CD8 ratio, duration of ART and type of ART regimen in our study. This is in contrast with other studies which demonstrated that RNFL thinning was associated with lower CD4 counts, longer ART duration and type of ART (18). Meanwhile, high viral loads were found to be associated with thicker RNFL (19). Cheng et al. also demonstrated that low CD4 count levels (<100 cells/μl) and reduced RNFL thickness was a significant predictor of impaired driving ability (36).These parameters were likely less important in our cohort where all participants were virologically suppressed on ART and a significant majority had achieved a CD4+ T-cell threshold of >500cell/ul.

The association between RNFL thickness and markers of immune activation were inconsistent in the cohort with associations in the CD8+ T-cell compartment being the most prominent across multiple RNFL quadrants. Even so, CD8+ T-cell activation, which was significantly higher in PLWH compared to controls, was associated with thickening and not thinning of RNFL and contrary to our expectation. The reason for this is not entirely clear though it is likely that there are multiple aetiologies contributing to changes in RNFL thickness in this cohort, including poor metabolic control, chronic inflammation and age-associated changes among others. Thicker RNFL among PLWH with well controlled disease was reported in the AGEhIV cohort and the authors ascribed this to low-grade inflammation (37). Age related RNFL thinning can vary across the different quadrants (38, 39), and longitudinal changes to RNFL over time depends on factors such as ethnicity, refractive error and diabetic status (40–43). These factors were not controlled for in this cohort, and hence may account for the non-uniform trend seen with the biomarkers. The exact association between markers of immune activation and RNFL thickness is best clarified in a longitudinal cohort. The cross-sectional nature of the study design precludes any temporal relationship we can infer from RNFL thinning and PLWH.

## Conclusion

Significant thinning of the temporal RNFL quadrant is seen in our cohort of PLWH on ART. This structural loss is reflected functionally as deficits in contrast sensitivity. These changes did not have any association with HIV clinical parameters or treatment, but was associated with diabetes. As the life expectancy of PLWH increases, the long-term implications of these findings on the PLWH’s visual functions and risk of visual impairment needs to be elucidated.

## Data availability statement

The raw data supporting the conclusions of this article will be made available by the authors, without undue reservation.

## Ethics statement

The studies involving humans were approved by University of Malaya Medical Research Ethics Committee. The studies were conducted in accordance with the local legislation and institutional requirements. The participants provided their written informed consent to participate in this study.

## Author contributions

MN, NN, NR, and RR wrote the article. RR, NR, SK, and AK conceived and supervised the study. MN, NSAB, YSH, and NSL contributed to data acquisition. MN, NR, and RR interpreted the data. MN and NN performed the statistical analysis. All authors contributed to the article and approved the submitted version.
